# Stuck in Time: Negative Income Shock Constricts the Temporal Window of Valuation Spanning the Future and the Past

**DOI:** 10.1371/journal.pone.0163051

**Published:** 2016-09-15

**Authors:** Warren K. Bickel, A. George Wilson, Chen Chen, Mikhail N. Koffarnus, Christopher T. Franck

**Affiliations:** 1 Virginia Tech Carilion Research Institute, Roanoke, VA, United States of America; 2 University of Kentucky, Lexington, KY, United States of America; 3 Virginia Tech Department of Statistics, Blacksburg, VA, United States of America; Johns Hopkins School of Medicine, UNITED STATES

## Abstract

Insufficient resources are associated with negative consequences including decreased valuation of future reinforcers. To determine if these effects result from scarcity, we examined the consequences of acute, abrupt changes in resource availability on delay discounting—the subjective devaluation of rewards as delay to receipt increases. In the current study, 599 individuals recruited from Amazon Mechanical Turk read a narrative of a sudden change (positive, neutral, or negative) to one’s hypothetical future income and completed a delay discounting task examining future and past monetary gains and losses. The effects of the explicit zero procedure, a framing manipulation, was also examined. Negative income shock significantly increased discounting rates for gains and loses occurring both in the future and the past. Positive income windfalls significantly decreased discounting to a lesser extent. The framing procedure significantly reduced discounting under all conditions. Negative income shocks may result in short-term choices.

## Introduction

The psychological consequences of scarce resources endemic to poverty are of growing scientific and societal importance [1, 2]. Research findings suggest that a lack of resources along with the stress associated with scarcity may shift attention toward short-term needs, even at the expense of longer-term goals. Relative preference for immediate vs longer-term reinforcers can be understood through the process of delay discounting, which refers to the devaluation of reinforcers as a function of the delay to their receipt. Indeed, considerable evidence supports an association between poverty and greater discounting of delayed rewards (see [3], for a review, [4]).

Although this correlation between poverty and greater delay discounting has been well established, interpreting this relationship can be challenging. That is, a preexisting preference for the short-term could result in choices that lead to poverty or, alternatively, poverty may produce a constriction of time preferences and reduce the valuation of future opportunities [2]. These competing hypotheses can be tested by the experimentally manipulated income loses and gains. Haushofer, Schunk, and Fehr [5], for example, manipulated losses and gains in income and relative income rank under experimental conditions. Specifically, participants began the experiment endowed with a large (rich) or small (poor) number of points that were later redeemable for money at the experiment’s end. The subjects were then exposed to a task in which they could earn or lose points and were informed of their rank earnings relative to others. During that task, the group that was richly endowed experienced an abrupt negative income shock that brought their point totals to the same level as the poorly endowed group. Subsequent measurement of delay discounting revealed that the group that experienced the negative income shocks along with reports of relative ranking among peers discounted future monetary rewards at a higher rate than those who were poorly endowed from the beginning. Positive income shocks weakly decreased rates of discounting of future monetary gains under analogous conditions. Thus, a negative income shock along with relative rankings among peers changed the valuation of future monetary gains, while positive income shock along with relative rankings produced marginal effects. However, whether those results are due solely to income shocks or to changes in relative ranking, or both, remains unclear.

Consistent with the predominate view that temporal discounting is a process that is associated with a linear, unidirectional conception of time, this prior study examined only inter-temporal choices for future monetary gains [6]. Accruing evidence, however, suggests that temporal discounting can be viewed as a temporal window that extends from the past into the future [6, 7]. For example, cigarette smokers and control participants discount future and past monetary amounts symmetrically, whether those amounts are gains or losses (see also [8]). Interestingly, the cigarette smokers discount both future and past monetary amounts more than controls.

Subsequent research supported the temporal window hypothesis of delay discounting by addressing the mechanism of the explicit-zero phenomenon, a framing effect [6]. The explicit-zero phenomena refer to how preferences shift toward larger delayed rewards when the implicit zero in discounting choices (i.e., “Would you prefer $80 now or $100 in a month?”) are reframed to make the implicit zero explicit (i.e., “Would you prefer $80 now and $0 in a month or $0 now and $100 in a month?”) [9]. Prior studies of the explicit zero effect with delayed discounting of future rewards have explained the increased valuation of larger later monetary amounts as a result of a preference for a sequence of increasing future positive outcomes. This hypothesis suggests that adding similar framing to the past discounting procedure would engender greater preference for the most recent option as opposed to the option that is further in the past, producing an interaction between explicit-zero effects on past and future discounting. Alternatively, the temporal window hypothesis would suggest that the explicit zero framing would symmetrically extend past and future discounting. The test of these hypotheses supported the temporal window hypothesis showing explicit zero framing resulted in a symmetrical preference shift toward the option further in the past and further into the future [6]. Whether the framing effect will still be observed when participants are experiencing an abrupt income shock is unknown,

Research in other areas has shown that narratives describing economic poverty can induce changes in fluid intelligence, cognitive control and racial perception, [10–12], but those studies did not include measures of future or past discounting of gains or losses. If the negative income shock narrative increases the rate of discounting into the past and future for both gains and losses, then such findings would be consistent with the view that poverty engenders greater discounting and that the effects of poverty are more extensive and complex than previously thought. Thus, we tested whether reading a narrative describing a negative income shock, relative to a neutral narrative, results in greater discounting of both future and past monetary gains and losses, consistent with a constricted temporal window. We also tested whether a narrative describing a positive income shock would produce effects opposite to those of negative income shock. Finally, we examined whether the explicit-zero effect differs across the different income narratives and discounting measures.

## Materials and Methods

### Participants

Six hundred participants were targeted for enrollment via Amazon Mechanical Turk (mTurk). For the six combinations of valence and framing (see Procedures), this sample size would afford 80% power to detect an Eta-square value of 0.0212, the type of effect expected and a small-medium sized effect by convention [13]. *N* = 599 participants were actually recruited. Eligibility was restricted to individuals who resided in the United States; were over the age of 18; and were not, nor had ever been, employed by Virginia Tech or the Virginia Tech Carilion Research Institute. Data from four subjects were excluded because they gave answers to subsequent demographic questions that were either not interpretable or inconsistent with the exclusion criteria. Subjects were paid $0.05 for initially accepting the HIT, and $4.95 for successfully completing the survey.

Participants were randomly assigned to one of six conditions, which combined different income narratives and implicit-/explicit-zero framing in the discounting tasks: (1) negative narrative with implicit-zero framing (n = 102), (2) negative narrative with explicit-zero framing (n = 101), (3) neutral narrative with implicit-zero framing (n = 98), (4) neutral narrative with explicit-zero framing (n = 98), (5) positive narrative with implicit-zero framing (n = 99), and (6) positive narrative with explicit-zero framing (n = 97). Analysis of demographic characteristics (gender, age, employment, income, marital status, and education) revealed no statistically significant differences between narrative conditions. Thirty-six percent of the participants were female. The mean age was 28.61 ± 8.41 (SD). The frequencies (percentages) of employment status were as follows: Full-time: 237 (39.83%), Part-time: 87 (14.62%), Self-employed: 76 (12.77%), Unemployed: 77 (12.94%), Retired: 3 (0.50%), Students: 113 (18.99%), and Other: 2 (0.34%). Income was highly skewed (skew = 6.12) with median $42,000 per year and interquartile range $25,000-$65,000.

### Procedures

All procedures in this study were reviewed and approved by the Virginia Tech Institutional Review Board. Participants, after meeting eligibility criteria, read the consent statement and accepted the Human Intelligence Task (HIT). A HIT is a small task for human workers to complete which is posted on Amazon Mechanical Turk, a crowdsourcing internet marketplace. Participants provided answers to demographic questions (see demographics above) and were then presented with the following instructions:

“In this experiment, you will be asked to first read and envision yourself experiencing a scenario for 15 seconds. You will then be given four surveys. During the surveys you will be repeatedly given two options and asked to select one assuming the scenario had happened to you. In two of the surveys you will be asked whether you would rather GAIN a hypothetical amount of money in either the future or the past. In the other two surveys you will be asked whether you would rather LOSE a hypothetical amount of money in either the future or the past. There are no right or wrong answers.”

The participants were then presented with their randomly assigned narratives (positive, neutral, or negative; see Table 1). When presented, the narrative remained on the participant’s computer screen for 60 seconds before they could continue the task to ensure that the participant read the narrative.

**Table 1:** Negative, neutral, and positive income narratives.

Narrative Valence	Narrative
Negative	You have just been fired from your job. You will now have to move in with a relative who lives in a part of the country you dislike, and you will have to spend all of your savings to move there. You do not qualify for unemployment, so you will not be making any income until you find another job.
Neutral	At your job, you have just been transferred to a different department in a location across town. It is a similar distance from where you live so you will not have to move. You will be making 2% more than you previously were.
Positive	At your job you have just been promoted. You will have the opportunity to move to a part of the country you always wanted to live in OR you may choose to stay where you are. Either way, the company gives you a large amount of money to cover moving expenses, and tells you to keep what you don’t spend. You will be making 100% more than you previously were.

The participants were then asked four different types of delay discounting questions in which they selected between two outcomes that involved: 1) gaining money in the future, 2) losing money in the future, 3) having gained money in the past, or 4) having lost money in the past. Specifically, the 5-trial adjusting delay discounting procedure was employed [14]. The sum of money closest to the present moment (either in the past or the future) was always $500 dollars, whereas the sum of money farther away from the present moment (again, either in the past or the future) was always $1,000. Across trials, participants’ choices titrated the delay to the larger option. From the participant’s answers, their discount rate was ascertained for each task (see [14], for a more detailed explanation of the procedure). As a manipulation check and to test narrative valance, participants were asked to rate their current mood on a seven-point Likert scale ranging from “very sad” to “very happy” after reading the narratives but before any discounting tasks were initiated (See Table 1 for the questions).

### Statistical methods

The discounting procedure used here provides a measure of the effective delay that results in a reduction in the subjective value of the discounted commodity by 50% (ED50). In order to render the data comparable to the vast majority of delay discounting data, the ED 50 value was converted to the discounting parameter *k* by inverting ED50 [15]. The discount rate parameter, *k* was natural log transformed [16]. The primary objective of the statistical analysis was to evaluate effects of income narrative (positive, neutral, negative) and framing (explicit zero, implicit zero) on discounting rate, *ln(k*,) in four conditions (future gain, future loss, past gain, and past loss) after identifying and accounting for other variables’ association with discount rate. The dependent measure of discounting was a natural log transformation of the discount rate parameter, *k* [16]. A linear mixed-effect modeling framework was used in conjunction with model selection in this analysis. The mixed-effects model accounts for repeated discounting measurements on subjects by including random effects term for subjects. The model selection approach (based on the Bayesian Information Criterion, BIC [17] selects those variables, among a list of candidate predictors, that should be included in a parsimonious model. To allow for the possibility that the effects of some predictors of discounting depend on the level of other predictors, statistical interaction terms were included in the analysis. The gains/losses comparison was a within-subject factor in this study so this comparison was made within the mixed model framework using contrasts. Significant statistical interaction between gains/losses and past/future conditions was observed (see Results), so contrasts were constructed to compare gains versus losses within the past condition and future condition separately.

### Model selection via Bayesian Information Criterion

Backwards selection was implemented using BIC to determine which subset of predictors to include in final models. Briefly, BIC measures the likelihood of a given model with a penalty for complexity (i.e., number of predictors), with lower values of BIC corresponding to models that are more parsimonious. A model is said to be more parsimonious if it adequately characterizes the discounting outcome without being overly complex. Backwards selection was used to search available models, with BIC as the criterion to determine the final model. Backwards selection proceeds by initially including all available candidate predictors, fitting the model, computing BIC, and then iteratively removing variables in the order that most reduces BIC until no further reduction in BIC is observed by removing any remaining variables. To assess statistical significance of model terms, p-values are presented based on the final model. Note that these p-values are computed with respect to the selected model and do not incorporate uncertainty associated with the model selection procedure. The value of predictors was established by their inclusion in models on the basis of BIC and their Eta-square effect sizes for this study. The p-values are a secondary measure provided for reference purposes only.

Candidate predictors of discounting *ln(k*) included income shock, gain/loss, framing, task order, age, gender, income, student status, employment status, and all possible two-way interactions of these predictors.

### Modeling methodology

Backwards selection began with all candidate variables and interactions included in the model. Each mixed model under consideration takes the following form:

$$y_{ij}=\beta_{0}+\beta_{1}x_{1ij}+\cdots +\beta_{p}x_{pij}+\gamma_{j}+\epsilon_{ij}$$ (1)

where *y_ij_* represents *ln(k*) for task *i* and subject *j*, *β*_0_ is an intercept, the *β*_1_,…,*β_p_* terms represent parameters corresponding to the candidate predictors *x*_1*ij*_,…,*_pij_*, *γ_j_* is a random effect for each subject such that $\gamma_{j}∼N(0,\sigma_{S}^{2})$, *ϵ_ij_* ∼ *N*(0,*σ*^2^) is an error term, and *i* = 1,…,4,*j* = 1,…,595, and *p* represents the number of predictors in the model. Categorical variables are incorporated in this analysis as a series of columns of zeroes and ones corresponding to levels of the variable, and statistical interactions are formed as the product of these columns. Restricted maximum likelihood was used to estimate parameters and perform inference in the mixed model framework.

The combined variable selection and modeling strategy terminated with the inclusion of statistical interaction between gain/loss and outcome time (past/future, see Results). Further analysis was considered within each of these four discounting types separately. This approach removes *γ_j_* and the *j* index from (1) and uses linear models without mixed effects for subsequent analysis. Least squares methodology was used to estimate parameters and perform inference for these models, and variable selection proceeded as described above in all four linear models. This strategy is similar to performing simple effects analysis, but yields models that are easier to interpret. Post-hoc assessment of model effects was conducted via model contracts with a Tukey adjustment to control for multiple testing contrasts. Normality of residuals was assessed graphically for all models. Eta-square effect sizes were computed for each model term. The Eta-square effects measure the proportion of variability attributable to each predictor while controlling for other model terms, making them analogous to *R*^2^ for individual model terms. Terms with a higher Eta-square account for more variability in discounting than terms with a lower Eta-square while controlling for all variables in the model. JMP Pro 11 was used for summary statistics, variable selection, and modeling *ln(k*) values. R version 3.2.0 was used for graphics. Generation of Eta-square effect sizes and pairwise comparisons were performed in SAS version 9.3. To determine whether the income shock affected participants, the scores from the emotion scale (see measures above) were analyzed using one-way ANOVA.

## Results

BIC backwards selection began on the full mixed model (1). The process terminated while a two-way interaction between past/future and gains/losses remained in the model. Further, significant interaction was observed between past/future and gains/losses in the presence of no other covariates (F = 28.99, DFNum = 1, DFDen = 1763.3, P-value < .001), the covariates listed above (F = 29.09, DFNum = 1, DFDen = 1760.3, P-value < .001), or the covariates listed above and their two-way interactions (F = 27.25, DFNum = 1, DFDen = 1716.3, P-value < .001). Hence, further results are reported by discounting type using linear models and least squares as described in the Methods section. Covariates, Eta-square effect sizes, and p-values for each type of discounting rate are summarized in Table 2. Covariates that are not shown in the table were excluded during the process of model selection.

**Table 2:** F test and semi-partial Eta-square results for each discount type.

	Future gain		Future loss		Past gain		Past loss	
Source	Eta-Square	p-value	Eta-Square	p-value	Eta-Square	p-value	Eta-Square	p-value
Narrative valence	0.0986	< .0001	0.0558	< .0001	0.0851	< .0001	0.0696	< .0001
Framing effect	0.0134	0.0029	0.0199	0.0003	0.0124	0.0041	0.0243	< .0001
Employment					0.0191	0.0468		
Order			0.0281	0.0004	0.0119	0.0475		
BIC	2572.3		2677.9		2802.6		2991.1	

### Effects of Income Shock on Delay Discounting

**Fig 1:**
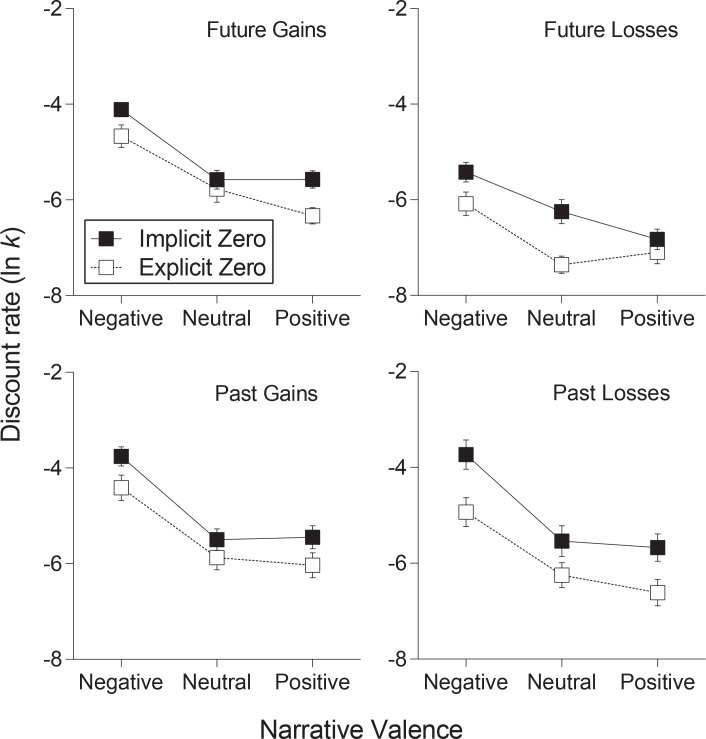
Mean discount rate in response to narrative valence for future. Gains (top left), future losses (top right), past gains (bottom left), and past losses (bottom right). Data are stratified by implicit-/explicit-zero framing. Error bars represent standard error of the mean.

Income shock was selected in all four types of discounting models, and in each case it had the largest Eta-square effect size of all included predictors (9.86% for future gain, 5.58% for future loss, 8.51% for past gain, and 6.96% for past loss, Table 2). Fig 1 displays bar plots for the means plus and minus one standard error with categories on the horizontal axis that correspond to narrative valence. This plot shows that *ln(k*) values indicative of steep discounting were evident in the negative income narratives relative to neutral and positive, with little evidence of differences between neutral and positive narratives in terms of l *ln(k*). These observations are corroborated in Table 3, which shows the significant differences between negative and neutral (*p* < .001 for all comparisons) and negative and positive (*p* < .001 for all comparisons) conditions. The estimated shifts in *ln(k*) ranged from 1.06 to 1.81 when comparing negative condition to neutral and positive across all four discounting conditions. There was no statistical evidence of differences between neutral and positive narratives in this study as evidenced by much smaller estimated shifts in lnk and non-significant p-values (Table 3).

**Table 3:** Pairwise comparisons between different levels of narrative valence and framing effect adjusted by other selected covariates (see Table 2). Results presented as: estimated shift (p-value). Estimated shift is in lnk. Bolded values represent statistical significance.

	Level	-Level	Future gain	Future loss	Past gain	Past loss
Narrative	negative	neutral	**1.29 (< .001)**	**1.06 (< .001)**	**1.53 (< .001)**	**1.56 (< .001)**
Valence	negative	positive	**1.56 (< .001)**	**1.23 (< .001)**	**1.63 (< .001)**	**1.81 (< .001)**
	neutral	positive	0.28 (>.250)	0.17 (>.250)	0.09 (>.250)	0.25 (>.250)
Framing effect	implicit	explicit	**0.51 (0.003)**	**0.66 (< .001)**	**0.57 (.004)**	**0.95 (< .001)**

### Effects of Explicit Zero on Delay Discounting

The framing effect on discount rate in each of the four models was significant (see Table 2), with Eta-square values of 1.34% for future gain *ln(k*), 1.99% for future loss *ln(k*), 1.24% for past gain *ln(k*), and 2.43% for past loss *ln(k*). Fig 1 shows that the implicit-zero condition produced higher discount rates in all four types of tasks. Table 3 indicates the estimated shifts in discount rate for these effects.

The mixed model p-values comparing gains to losses was statistically significant for both the future and past conditions (p<0.0001 for both). Fig 1 reflects this result, with gains discounted significantly more in both future and past conditions.

### Other Associations with Delay Discounting

Table 2 indicates that employment and order effects survived backward model selection in certain discounting models. Employment was included in the past gain model with an Eta-square effect size of 1.91%. Order was included as a main effect in future loss (Eta-square = 2.81%) and past gain (Eta-square 1.19%) models. Our model selection approach suggests these terms have a unique capacity to predict *ln(k*) in addition to valence and framing effects, thus they were included as covariates for these models. Hence, these effects were statistically accounted for in Tables 2 and 3 and Fig 1.

### Manipulation check

The ANOVA for the manipulation check revealed that mood following exposure to negative, neutral, and positive income shock narratives all differed significantly from each other (F = 265.17, DF_num_ = 2,DF_den_ = 592,p<0.0001, R^2^ = 47.25%). Further pairwise comparisons revealed that the negative (n = 203, mean = 2.82, SE = 0.09), neutral (n = 196, mean = 5.03, SE = 0.09), and positive (n = 196, mean = 5.44, SE = 0.09) narratives all differ individually (p = 0.003 for neutral versus positive, and p<0.001 for neutral versus negative and positive versus negative).

## Discussion

In this experiment, we showed for the first time that reading a narrative describing abrupt negative income shock increases discount rate, relative to a positive or neutral narrative, for both past and future monetary gains and losses. Reading a positive income narrative, in contrast, appears to have no effect on discount rate relative to either of the other narratives. Moreover, compared to the neutral narrative, negative and positive narratives significantly worsen and improve mood, respectively. However, the negative narrative appears to produce a much larger effect on mood. We discuss four points regarding our findings below.

First, the present findings are consistent with, and extend the observations of, Haushofer et al. [5], who found that negative income shock and changes in income rank among peers increased discount rate for future monetary gains and worsened mood. Here we report that reading a narrative describing negative income shock similarly increased discount rate for future monetary gains and worsened mood, but also extend those observations to discounting of future monetary losses, past monetary gains, and past monetary losses. Importantly, we show these effects without explicitly informing participants of their income rankings among peers. These findings support the hypothesis that income reduction can result in greater past and future discounting. Moreover, our findings are consistent with the view income shock may constrict the temporal window of valuation that extends from the past into the future. This constriction of the temporal window is consistent with evidence using a variety of different methods showing that material scarcity produces performance decrements and attentional deficits [10–12]. However, other interpretations of our data are possible including the narrowing of attention and or the result of framing. Moreover, the marginal effect of the positive income shock narrative replicates the findings of Haushofer et al., [5] and is consistent with prospect theory, which suggests that losses have greater effects than comparable magnitude gains [18]. In this study we attempted to match the two narratives. Prospect theory would suggest to get comparable magnitudes of effect would require considerable greater magnitude of positive relative to the negative narrative.”

Second, our findings may contribute to the understanding of the consequences of precipitous loss of income such as that experienced during the beginning recession that began in 2008. Here, the evidence suggests that negative income shocks may render individuals as more “stuck in time” [19, 20], in which they discount the past steeply and inadequately anticipate the future. These effects, in turn, may lead to further economic misfortune. An important future question for research in this area is how the time-course of this effect in the laboratory context relates to what is experienced in the real-world economy.

Third, our results replicate previous findings regarding explicit zero, [6], and differences in discounting between gains and losses [9, 21], and the utility of the specific discounting paradigm used [14]. Framing the choice in terms of both the gains and losses associated with making a choice between a sooner or later option generally produced a greater preference for the delayed options across conditions. This effect then appears not to interact with, or be constrained by, negative or positive income shocks. Thus, framing questions in this manner may serve to “nudge” greater preference for delayed outcomes, and may be added to the armentarium of choice architecture [22]. Previous studies have shown that losses are discounted less than gains. Here we replicate this so called “sign effect” which is consistent with the prospect theory showing that monetary losses are discounted less than monetary gains. Finally, the current study further validates and demonstrates the utility of adaptive discounting procedure employed here [14], which determines in only 5 choices the delay interval that is associated with indifference between the larger, delayed amount and the smaller, immediate amount. This measure of half-life is the inverse of the discount rate parameter, *k*. Thus the discount parameter can be determined quickly, making the procedure useful in future studies that seek to investigate conditions, such as income shock, that induce sudden and dynamic changes in reward preference. Replicating both the explicit zero effect and the sign effect demonstrates that this procedure provides result consistent with other procedural variants.

Fourth, among this study’s weaknesses include the use of hypothetical monetary amounts, as opposed to real, in the discounting task and in narrative of monetary gain and loss. Although our methods did not examine actual monetary outcomes, previous studies have shown comparable results when comparing real and hypothetical outcomes both behaviorally and neurally and have shown significant correlations between hypothetical discounting and actual monetary behavior [23, 24]. Also the scarcity narratives employed here did not actually manipulate income gains or losses, but our results are comparable to the results of the prior study that manipulated actual gains and losses.

In conclusion, the results confirm that greater temporal discounting can result from negative income shock and show that this effect is not only associated with greater discounting of future monetary gains, but also produces greater discounting of past monetary outcomes (i.e., gains and losses). These two effects may, in turn, lead to disadvantageous decisions. Research has shown that scarce economic resources are associated with increases in impulsivity and difficulty in problem solving. The framing of the explicit zero in the present study improved preference for delayed outcomes even in the presence of the negative income shock narrative and may suggest its utility in other contexts. The explicit-zero framing effect, however, was independent of the effects of negative income shock, as we observed no interaction between effects. Identifying whether the interventions can protect individuals from the negative consequences of poverty may be a worthwhile goal for future research.

## Supporting Information

S1 Dataset Raw data set used for analyses in this manuscript. (CSV)
